# In dogs with thoracolumbar disc extrusion does the use of acupuncture improve clinical recovery?

**DOI:** 10.18849/ve.v9i2.684

**Published:** 2024-05-22

**Authors:** Justin Ng+

**Keywords:** ACUPUNCTURE, CANINE, DOGS, ELECTROACUPUNCTURE, INTERVERTEBRAL DISC EXTRUSION, IVDE

## Abstract

**PICO question:**

In dogs with thoracolumbar intervertebral disc extrusion does the use of acupuncture with medical management compared with medical management alone improve clinical recovery?

**Clinical bottom line:**

**Category of research:**

Treatment.

**Number and type of study designs reviewed:**

Three papers were critically appraised: one randomised controlled trial, one non-randomised controlled trial, and one cohort study.

**Strength of evidence:**

Moderate.

**Outcomes reported:**

Acupuncture, and more specifically the combination of electroacupuncture and manual stimulation of acupuncture points when used as an adjunct to medical management, is more likely to result in both the recovery of ambulation and a quicker recovery of ambulation in dogs presenting with nonambulatory paraparesis or paralysis with deep pain perception due to thoracolumbar intervertebral disc extrusion, compared with medical management alone. It is less likely to make a difference in dogs that present with paralysis and no pain sensation.

There is less robust evidence supporting the use of bee venom injections in acupoints, however; it too may have a beneficial effect when used as an adjunct treatment in dogs with nonambulatory paraparesis or paralysis with deep pain perception due to thoracolumbar intervertebral disc extrusion, compared with medical management alone.

**Conclusion:**

There is moderate evidence supporting the conclusion that there is a mild benefit in the use of acupuncture with medical management to improve the clinical recovery of dogs with thoracolumbar intervertebral disc extrusion.

## How to apply this evidence in practice

The application of evidence into practice should take into account multiple factors, not limited to: individual clinical expertise, patient’s circumstances and owners’ values, country, location or clinic where you work, the individual case in front of you, the availability of therapies and resources.

Knowledge Summaries are a resource to help reinforce or inform decision-making. They do not override the responsibility or judgement of the practitioner to do what is best for the animal in their care.

## Clinical scenario

An 18-month-old Dachshund presents to your clinic with acute ataxia and nonambulatory paraparesis. After history taking, performing both a physical and neurological examination, you localise the lesion to the T3–L3 region in the spinal cord, with a high index of suspicion for intervertebral disc extrusion (IVDE). You recommend referral to a neurologist for a magnetic resonance imaging scan to reach a definitive diagnosis and to guide treatment, but that option is not financially viable for the client. You then recommend strict rest for 4–6 weeks and analgesia but wonder if acupuncture may be used as an adjunct to assist in the clinical recovery of the patient.

## The evidence

One randomised controlled trial was found (Hayashi et al., 2007), which in the hierarchy of evidence ranks near the top as a guide for decision-making about treatment efficacy. Another controlled trial (Tsai et al., 2015) documents that allocation of subjects was random, but then describes a deterministic method of allocation, making it more likely to be a non-randomised controlled trial. The third paper is a cohort study (Han et al., 2010). The three papers collectively should have provided strong evidence, however, there are significant issues which limit the strength of evidence and consequently the conclusions of the studies.

Categorisation of injury severity in patients with intervertebral disc extrusion (IVDE) is usually in reference to the modified Frankel scale (MFS) as described in a canine study (Levine et al., 2006). None of the studies used the MFS to assess outcomes, but the descriptors used allowed for categorisation of subjects into MFS grades. Thus, to allow for comparison of outcomes across the studies, the author of this Knowledge Summary has assigned grades to the subjects according to the MFS: grade 1 is defined as spinal pain only, grade 2 as ambulatory paresis, grade 3 as nonambulatory paresis, grade 4 as paralysis with deep pain perception and grade 5 as paralysis with no pain sensation. The MFS grade is presented in italic font within the summary of evidence tables.

The study's authors categorised treatment groups in a non-standardised fashion. As such, to allow for greater ease of comparison, in the summary tables, the treatment groups were standardised such that group M received medical management and group A received acupuncture as an adjunct alongside medical management. This is presented in italic font to differentiate from the original groupings in the respective papers.

## Summary of the evidence

**Han et al. (2010):** 

Population	Paraplegic dogs with intact deep pain perception (Grade 4) were treated during 2001–2005 at Konkuk University, South Korea, and two local animal clinics. (The modified Frankel scale grade is presented in italic font throughout the evidence table). Diagnosis of thoracolumbar intervertebral disc herniation was confirmed through myelography or magnetic resonance imaging. Additional tests (blood, radiography, and cerebrospinal fluid analysis) were performed to differentiate disc herniation from other conditions. A retrospective review of medical records was conducted.
Sample Size	80 dogs.
Intervention Details	Dog regimens were determined based on veterinary consultation, owner preference, and financial status. Dogs were strictly confined for at least 2 weeks, allowing for only urination and defaecation. Dogs received either conventional medicine alone (Group C / *Group M*) or a combination of conventional medicine along with electroacupuncture and traditional Chinese acupuncture (Group CE / *Group A*). (The grouping used by the author of this Knowledge Summary is presented in italic font throughout the evidence table). Dogs were allocated to *Group M* or *A* following a discussion with a veterinarian and considering the owner's preferences and financial situation. *Group M *(n = 37): Prednisolone (1 mg/kg, for 3 days; then 0.5 mg/kg, for 2 days; then 0.25 mg/kg, for 2–7 days) and cimetidine (10 mg/kg, for 7–12 days), all given every 12 hours and per os. Additional medications were given as needed (if subjects experienced gastrointestinal problems or urinary tract infections). *Group A *(n = 43): Same medical treatment as *group M* with acupuncture (electroacupuncture and traditional Chinese acupuncture). Acupuncture involved stainless steel needles (0.3 x 40 mm) inserted to a depth of 0.5–1 cm perpendicularly. Acupuncture was performed three times a week for 1–4 weeks. Electrostimulation was applied for 25–30 minutes a day, at 0.5–2.5 mV, with frequency alternating between 2 Hz x 5 sec and 15 Hz x 3 sec, adjusting for the dog’s comfort. Acupoint selection: Electroacupuncture: based on recovery period and lesions. Traditional Chinese acupuncture: performed on local (urinary bladder meridian) and distal (gall bladder and stomach meridian) points.
Study design	Cohort study.
Outcome studied	Subjective assessment: The owners were asked to describe gait. It was unclear when this assessment was carried out. For each treatment group, the percentage of dogs that had a successful recovery of gait, defined as the ability to walk without assistance (achieved either *grade 1* or *2)*. Time to recover ambulation (achieve either *grade 1* or *2)*. To investigate dogs that experienced recurrence of nonambulation (*grade 4* or *5*), further assessment was performed 1–4 years (mean 2.4 years) post treatment.
Main findings (relevant to PICO question)	A significant difference (P = 0.01) was detected in the percentage of dogs (25/37 [68%] in *group M* and 39/43 [91%] in *group A*) that successfully recovered their gait (*grade 1* or *2)*. There was a significant difference (P = 0.011) between the time (18.48 ± 0.88 days in *group M* and 15.15 ± 0.84 days in *group A*) (mean ± standard error) to recover ambulation (*grade 1* or *2)*. A significant difference (P = 0.031) was detected in recurrence rate between the percentage of dogs that had recurrence of nonambulation (*grade 4* or *5)*)between treatment groups (9/25 [36%] in *group M* and 5/39 [13%] in *group A*)*.* 16/80 dogs were excluded from this assessment due to requiring additional treatment with surgical decompression or an unsuccessful recovery during treatment. *Group M* dogs were monitored 3–14 months after treatment and *group A* dogs were monitored 3–38 months after treatment. Other findings include breed, body weight, age, spinal lesion neurolocalisation, number of lesions, complications, and other neurological deficits not defined within the modified Frankel scale (MFS). These do not relate to the PICO question and therefore will not be commented on further in this Knowledge Summary.
Limitations	Unclear if the outcome measure (time to recover ambulation [*grade 1* or *2*])was measured in days after treatment completion, potentially creating a discrepancy between the treatment groups. Missing specifications on diagnostic modalities and tests performed and potentially flawed diagnosis when using imaging modalities with lower sensitivities. The study did not consider any sort of pain management in the conventional medicine group, falling below the minimum standard of care expected in veterinary medicine. This influenced the difference of period of relief of back pain between the two groups. The current evidence for medical management of intervertebral disc extrusion (IVDE) supports the use of nonsteroidal anti-inflammatory drugs rather than prednisone. There are no details on the bladder management of the dogs in this study, falling below the minimum standard of care expected in veterinary medicine. Absence of details on the study period. Lack of differentiation between recruitment, exposure, follow-up, and data collection periods. No description of animal housing or location of care. No reporting on caregivers’ eligibility criteria. Lack of information on follow-up methods and blinding. Absence of data accuracy assessment. Absence of description regarding the causal structure among variables. Failure to describe the development, validation, and administration of the owner assessed outcomes. Failure to address bias (for instance bias is introduced when two dogs treated with surgical decompression ‘later’ were excluded), confoundin,g and missing data. Did not state whether treatment groups were selected* a priori* or based on the collected data. Insufficient consideration of potential confounders (e.g. single or multiple disc lesions). No adjustment for multiple hypothesis testing. Absence of robustness analyses or transparency in eligibility. Did not report the number of owners at any level of the study. Incomplete reporting of outcome assessments. Missing outcome variability estimates and precision measures. Unclear distinction between pre hoc and post hoc analyses.

**Hayashi et al. (2007):** 

Population	Dogs with signs of thoracolumbar intervertebral disc disease (IVDD) were evaluated from March 2005 to February 2006 at the veterinary hospital of the University of São Paulo, Brazil. The severity of IVDD ranged from having no neurologic signs except pain associated with IVDD (*grade 1*), conscious proprioceptive deficit and ambulatory paraparesis (*grade 2*), nonambulatory paraparesis and deep pain perception (*grade 3*), nonambulatory paraplegia and deep pain perception with or without urinary dysfunction (*grade 4*) to nonambulatory paraplegia and no deep pain perception with or without urinary dysfunction (*grade 5*). (The modified Frankel scale (MFS) grade is presented in italic font throughout the evidence table).
Sample Size	50 dogs.
Intervention Details	Owners were advised on special care for dogs with urinary retention or paralysis, including preventing skin lesions, urinary tract infections, and restricting their activity for a minimum of 30 days. Dogs received either standard Western medical treatment combined with electroacupuncture and traditional Chinese acupuncture (Group 1 / *Group A*) or standard Western medical treatment alone (Group 2 / *Group M*). (The grouping used by the author of this Knowledge Summary is presented in italic font throughout the evidence table). Dogs were randomly allocated by lot to each treatment group. *Group M *(n = 24): There was one dog with *grade 1, *six with* grade 2, *one with *grade 3, *eight with* grade 4 *and eight with* grade 5*. Prednisolone (1 mg/kg, for 3 days; then 0.5 mg/kg, for 5 days; then 0.5 mg/kg, for 5 days) all given once daily and per os. Additional medications were given as needed (if subjects experienced gastrointestinal problems or needed additional analgesia). *Group A *(n = 26): There were two dogs with *grade 1*, eight with *grade 2*, three with *grade 3*, seven with *grade 4* and six with *grade 5*. Same medical treatment as *Group M* with acupuncture (electroacupuncture and traditional Chinese acupuncture). Electroacupuncture involved needles (0.25 x 25 mm). Electroacupuncture was performed 1–2 times a week for 3–4 weeks. Electrostimulation was applied for 20 minutes at a frequency that alternated between 3 Hz and 100 Hz. Acupoints were selected based on the acupuncturist’s clinical experience, literature, and traditional Chinese medicine.
Study design	Prospective parallel non-masked randomised controlled trial.
Outcome studied	Subjective assessment: Assessments took place once a week for at least 3 weeks. Number of dogs that recovered from *grades 3* and *4* to *grade 1* or *2* (assessed by owner or veterinarian). Time taken for this recovery to be achieved (assessed by owner or veterinarian). Number of dogs that recovered from *grade 5 *to *4 *(evaluated by a veterinarian).
Main findings (relevant to PICO question)	In *grades 3 and 4 *dogs: There was a significant difference (P = 0.0341) between the time (10.1 ± 6.49 days in *group A* and 20.83 ± 11.99 days in *group M*) (mean ± SD) taken to recover to *grade 1* or *2*. There was also a significant difference (P = 0.047) between the rate of dogs with *grades 3* and *4* (10/10 [100%] in *group A* and 6/9 [67%] in *group M*) that recovered to *grade 1* or *2.* There was no significant difference (P = 0.124) between the rate of dogs with *grade 5* (3/6 [50%] in *group A* and 1/8 [12.5%] in *group M*) that recovered to *grade 4.* Data from 61 dogs was initially evaluated but only 50 completed the study. Reasons for loss to follow-up were undefined. Partial to total urinary control was achieved in (8/10 [80%] in *group A* and 6/12 [50%] in *group M*). Other findings include sex, breed, body weight, age, duration of clinical signs before commencement of the study, functional numeric scale scores, time of final assessment, and other neurological deficits not defined within the MFS. These do not relate to the PICO question and therefore will not be commented on further in this Knowledge Summary.
Limitations	Omitted from the main findings: Recovery of *grades 1* and *2* included assessment of factors (signs of pain and conscious proprioception) that are outside the descriptors used in the MFS. No information on p-value for the difference in recovery time between treatment groups in *grade 5*. Ambulation recovery comparison between treatment groups included dogs that were already ambulatory. Uncertainty regarding the assessor of improvement in ataxia. For *grade 5 *dogs, the time to recover ambulation was defined as success but submitted only for descriptive statistical analysis due to a small number of dogs that regained ambulation. Unclear if the outcome measure (time to achieve a lower *grade*) was measured in days after treatment completion, potentially creating a discrepancy between the treatment groups. Missing specifications on diagnostic modalities and tests performed and potentially flawed diagnosis when using modalities with lower sensitivities. No reporting on caregivers’ eligibility criteria or whether animals were shelter or client owned. No description of animal housing or location of care. Potential variability in the prednisolone protocol due to pretrial medication. Lack of information on certain medication protocols. Lack of evidence for the effectiveness in the use of tramadol for pain control. Did not report the material, retention time, number of insertions, depth of needle insertions, or responses sought through acupuncture. Acupuncture points varied amongst group of dogs with no explanation for the different choices. No reason was given for the different frequency of acupuncture treatment used: i.e., from once a week for at least three applications vs twice a week for 2 weeks followed by once a week for 2 weeks for dogs without deep pain. No details on the current or amplitude of electrical stimulation. No description of participating acupuncturists qualifications. Unclear distinction between pre hoc and post hoc outcomes and analyses. Randomisation and blinding were inadequately described. Did not specify whether analysis was based on intention to treat or per protocol. Analysis later done per protocol without adjustment for pre and post randomisation prognostic factors. No description of the data form of the outcome measures. No indication of whether the study aimed to assess superiority, equivalence, or noninferiority. Failure to address the nonindependence between outcomes due to repeated measures over time. No description of the enrolment numbers, adherence to planned interventions, or loss to follow-up. Insufficient consideration of potential confounders (e.g. Hansen type I or II, single, or multiple disc lesions). No mention of the statistical analysis software used. Did not report the estimated magnitude of differences between groups (effect size), nor the precision (e.g. 95% CI). Missing outcome data (group means), variability estimates and precision measures. No description of methods for detecting adverse events or reporting their occurrence or absence. Subgroup analyses should have been split into *grade 3* and *grade 4* dogs (or equivalent) as these groups often have different outcomes with medical management alone.

**Tsai et al. (2015):** 

Population	Presumptively diagnosed thoracolumbar disc protrusion or extrusion based on clinical signs (evaluated by specialised veterinarians), history, and radiography. The severity of neurological deficits ranged from having focal pain only (*grade 1*), ability to bear weight, proprioceptive deficits and ambulatory paraparesis (*grade 2*), inability to bear weight, severe incoordination, intact spinal reflexes, or hyperreflexia, nonambulatory paraparesis, proprioceptive deficits, and deep pain perception (*grade 3*) to any of the aforementioned clinical signs in addition to paraplegia, no deep pain perception, and bladder dysfunction (*grade 5*). The modified Frankel scale (MFS) grade is presented in italic font throughout the evidence table. Myelography or computed tomography were performed when necessary for diagnosis. Study was conducted in three private animal hospitals in Taichung, Taiwan, between August 2010 and August 2012. Dogs were excluded if they had other comorbidities such as heart, liver or kidney failure, encephalitis, bacterial infection or viral infections, and other causes of neurological dysfunction.
Sample Size	40 dogs.
Intervention Details	Dogs received either standard medical treatment (control group / *Group M*) or a combination of standard medical treatment along with bee venom injection at acupoints (experimental group / *Group A*). (The grouping used by the author of this Knowledge Summary is presented in italic font throughout the evidence table). Dogs were assigned to each treatment group based on the order in which medical advice was sought. *Group M *(n = 20)*:* There were two dogs with *grade 1, *five with* grade 2, *five with *grade 3 *and seven with* grade 5*. Prednisolone (1 mg/kg/day, per os) and carprofen (2.2 mg/kg/day, administration route undefined), both for 7 days. Ranitidine (2 mg/kg/day, administration route undefined) for 5–7 days to prevent gastrointestinal disturbances. Additional antibiotics were given if subjects experienced a urinary tract infection. *Group A *(n = 20)*:* There was one dog with *grade 1, *four with* grade 2, *seven with *grade 3 *and five with* grade 5*. Same medical treatment as *group M* and bee venom injections at acupoints. Acupoints were selected based on traditional Chinese medicine. Injections were performed twice a week for 6 weeks. Bee venom injection solution contained: Apimellena (main ingredient: apitoxin) at 5 mg/vial diluted using 6.2 mL of saline, 0.4 ml of apitoxin (400 ㎍), and 0.4 ml of lidocaine (8 mg). Each acupoint was sterilised by 0.1 ml (equivalent to 20 ㎍) alcohol, prior to the injection.
Study design	Prospective parallel partially masked non-randomised controlled trial.
Outcome studied	Subjective assessment: Veterinarian who was blind to the grouping assessed each dog for neurological deficits. Assessment was performed before treatment and approximately on a weekly basis, including 2 weeks (14 ± 3 days) after treatment.
Main findings (relevant to PICO question)	In *grade 3 *dogs, there was a significant difference (P < 0.05) in neurological grade (MFS equivalent) between the treatment groups, 2 weeks after treatment. One dog in *group M* was withdrawn from the study. In *group A*, one dog was withdrawn, one died and one underwent surgery. Other findings include sex, age, weight, myelopathy scoring system grade, functional numeric scale score, and other neurological deficits not defined within the MFS. These do not relate to the PICO question and therefore will not be commented on further in this Knowledge Summary.
Limitations	Only one p-value was reported for the comparison between treatment groups thus other outcomes were omitted. Statistical data not documented with SD limiting the ability of the reader to calculate p-values for differences between group means. Unclear if the outcome measure (*grade* after treatment*) *was measured in days after treatment completion, potentially creating a discrepancy between the treatment groups. Intervention includes bee venom which may have had its own therapeutic effect. Unclear distinction between pre hoc and post hoc protocol, outcomes, and analyses. The study is jeopardised due to the lack of definitive diagnosis i.e., 'presumed thoracolumbar disc protrusion or extrusion based on clinical signs, history, and radiographic physical examination'. No reporting about caregivers’ eligibility criteria or whether animals were shelter or client owned. No description of animal housing or location of care. Did not state if any medication / treatment was prescribed prior to the trial. Lack of information on certain medication protocols and bee venom. Use of both prednisone and carprofen typically contraindicated. Minimal provision for reasoning for the chosen acupuncture treatment including rationale for diagnosis, point selection, and treatment procedures. Did not report the acupuncture needle type, retention time, stimulation techniques, duration of sessions, or depth of needle insertions. No description of participating acupuncturists' qualifications. No details on what 'specialised veterinarians' mean. The primary time point of interest in terms of outcome is not clearly identified. No reasons given for cases that were withdrawn from the study or died. Bias was introduced due to the withdrawal of one case that underwent surgery. Randomisation and blinding were inadequately described. Did not specify whether analysis was based on intention to treat or per protocol. Analysis may have given exaggerated assessment of treatment effect through a lack of adjustment for pre and post randomisation prognostic factors (done on a per-protocol rather than intention-to-treat basis). No description of the data form of the outcome measures. No indication of whether the study aimed to assess superiority, equivalence, or noninferiority. Failure to address the nonindependence between outcomes due to repeated measures over time. No mention of the statistical analysis software used. Did not state the number that were assessed for eligibility. Insufficient consideration of potential confounders (e.g. chondrodystrophic vs non-chondrodystrophic breed, Hansen type I or II, duration of clinical signs before entering the study, single or multiple disc lesions). Missing outcome data (group mean), variability estimates and precision measures. No description of methods for detecting adverse events or reporting their occurrence or absence. Subgroup analyses should have been split into *grade 3* and *grade 4* dogs (or equivalent) as these groups often have different outcomes with medical management alone. Data about reduction vs increment of functional numeric scale and myelopathy scoring system grades between groups could be more organised. Lack of explanation on what 'repair time' is and how this can be objectively assessed. Lack of details on how patients with incontinence were addressed.

## Appraisal, application and reflection

The controlled trials (Tsai et al., 2015; and Hayashi et al., 2007) and the cohort study (Han et al., 2015) were appraised in accordance with the PetSORT guidelines (Sargeant et al., 2023) and the STROBE-Vet statement (O'Connor et al., 2016), respectively. The outcomes reported across the studies surmise that acupuncture, electroacupuncture, and bee venom injections are more likely to result in both the recovery of ambulation and a quicker recovery of ambulation in *grades 3* and *4* modified Frankel scale (MFS) dogs but are less likely to make a difference in *grade 5* MFS dogs. The evidence supporting the use of acupuncture and electroacupuncture is marginally stronger than the evidence supporting the use of bee venom injections in acupoints; however, the evidence is overall moderate. The limitations that are especially pertinent are expanded on in the following paragraphs.

The three studies did not use a single unified scale when assessing clinical recovery. While both Tsai et al. (2015) and Hayashi et al. (2007) used functional numeric scale (FNS) in their assessments, this was not a validated scale in the assessment of neurological recovery. The MFS was chosen by the author of this Knowledge Summary over other scales validated for use in spinal cord injuries (Levine et al., 2009) because it is widely used in the assessment of dogs with intervertebral disc extrusion (IVDE), as stated in the American College of Veterinary Internal Medicine consensus statement (Olby et al., 2022). This did lead to several instances where the outcomes assessed in the studies were outside the scope of the MFS; for example, one of the ways Han et al. (2010) assessed clinical recovery was through improvement in urine control. In these instances, the outcomes did not relate to the PICO question and therefore were not commented on further in this Knowledge Summary.

A limitation in study design that was present in all three papers was the lack of clarity behind when clinical recovery was assessed; if it was after treatment was completed, then the timings could have a variation of up to 35 days between treatment groups (Table 1). Consequently, the extended duration of exercise restriction, that was part of the intervention in two of the studies (Han et al., 2010; and Hayashi et al., 2007), could have acted as a potential confounder, favouring the treatment group that included acupuncture. Interestingly, a study (Levine et al., 2007) of dogs with IVDE found that the length of cage rest enforced by the client had no impact on the outcome. The only outcome relevant to the PICO question that was explicitly stated to be compared at the same time point, was the one reported by Tsai et al. (2015) in the summary of evidence tables. This finding, however, is arguably not as clinically relevant as the main findings in the other two studies; it was not assessing a well-defined outcome, such as recovery of ambulation, but was instead assessing the difference in neurological grade between the two treatment groups.

**Table 1: Duration of each intervention:** 

Study	Medical group (days)	Acupuncture group (days)	Variation in timings (days)
Han et al., 2010	7–12	7–28	0–21
Hayashi et al., 2007	13	21–28	8–15
Tsai et al., 2015	7	42	35

Strictly speaking, the outcome assessed in two of the studies (Han et al., 2010; and Hayashi et al., 2007) was functional improvement (recovery of ambulation) as opposed to recovery of the pathological effects that occurred due to the extrusion of nucleus pulposus (Fadda et al., 2013). In fact, Olby et al. (2022) recommends a period of restricted activity for a minimum of 4 weeks, putatively aiming to facilitate healing of the annulus fibrous. Hence, even if acupuncture did result in a quicker recovery of ambulation, that may not necessarily have been in the patient’s best interests in terms of long-term outcome or recurrence. However, Han et al. (2010) demonstrated that quicker recovery of ambulation did not result in a higher rate of recurrence; there was a significantly (P = 0.031) higher rate of recurrence in the medical management group (9/25 [36%] dogs monitored 3–14 months after treatment) compared to the group that received acupuncture (5/39 [13%] dogs monitored 3–38 months after treatment).

Both Tsai et al. (2015) and Hayashi et al. (2007) classified subjects into subgroups by categorising subjects in both the equivalent of *grades 3* and *4* MFS together, on the basis on having similar FNS scores during their initial assessment. When comparing the outcomes of medically managed *grade 3* and *grade 4* MFS dogs, Olby et al. (2022) reports that 81% of dogs in *grade 3* will recover ambulation compared to 60% in *grade 4* MFS. Thus, by categorising dogs into this subgroup, there may have been an increased risk of type 1 error (Tukey, 1977) (created by the author of this Knowledge Summary due to the imposition of the MFS scale on the papers) in *grade 4* MFS dogs.

Another issue that all the papers had was having an excessive number of primary outcomes, which complicated the interpretation of results; there were different inferences for each outcome which may have led to issues of multiplicity in analyses (Sargeant et al., 2023). One such instance would have been that different sample size calculations were needed for each outcome. The post hoc power of each study based on the outcomes assessed in this Knowledge Summary were calculated (Kane, 2018), where possible (the post hoc power of the main findings in Tsai et al. (2015) could not be calculated due to the lack of figures provided), using a probability of type I error (α): 0.05 (Table 3). These ranged from 33–73.7%, which would mean that the studies were underpowered in certain instances, in comparison to the 80% power that most studies are set at (Charan & Kantharia, 2013).

**Table 3: Post hoc power:** 

Study	Outcome	Post hoc power (%)
Han et al., 2010	Recovery from *grade 4* to *grade 1* or *2* MFS	73.5
Han et al., 2010	Recurrence rate of *grade 4* or *5* MFS	57.9
Hayashi et al., 2007	Time to recover from *grades 3* and *4* to *grade 1* or *2* MFS	66.6
	Recovery from *grades 3 *and 4* to *grade 1* or *2 *MFS	50.4
	Recovery from *grade 5* to *grades 1–4* MFS	33.0

*The calculator would not allow for a study incidence to be 100%, so 99.9% was used instead.

The eligibility criteria for the subjects varied widely with regards to the approach in diagnosing IVDE (Table 4). Imaging has been omitted by Hayashi et al. (2007); while no evaluation of sensitivity could be found for only using clinical signs as a diagnostic approach, it is unlikely that examination alone can reliably differentiate IVDE from other spinal diseases. The sensitivities of the other utilised diagnostic modalities are listed (Table 5) to aid in their comparison. Tsai et al. (2015) and Han et al. (2010) did not specify the subjects that received each imaging modality. Hence, eligibility criteria for most of the subjects across the studies may have been based on a diagnostic modality with only low to moderate sensitivity.

**Table 4: Diagnosis of IVDE:** 

Study	Diagnosis
Han et al., 2010	Myelography or magnetic resonance imaging
Hayashi et al., 2007	Clinical signs
Tsai et al., 2015	History, clinical signs, radiography, myelography or computed tomography

**Table 5: Sensitivity of diagnostic approaches:** 

Study	Diagnostic approach	Sensitivity (%)
Cooper et al., 2013	Magnetic resonance imaging	98.5
	Computed tomography	88.6
Hecht et al., 2009	Myelography	78.9
Schulz et al., 1998	Radiography	60.0

In terms of documenting adverse events, only Han et al. (2010) reported harms, and even then, only accounted for vomiting and diarrhoea, which were more medicine centric, and did not assess for other more common adverse effects of acupuncture, such as peripheral nerve injury, as described in a systematic review in human medicine (Wu et al., 2015).  Acupuncture offers a relatively safe therapeutic option compared to other medical management options; a canine study (Baker–Meuten et al., 2020) that monitored for adverse effects of acupuncture demonstrated that there were none in the study.

Through the creation of this Knowledge Summary, it became evident to the author that there is a dearth of adequately published studies in the use of acupuncture in the treatment of canine IVDE. Furthermore, to the author of this Knowledge Summary’s knowledge, there is an absence of any canine studies comparing the differences in efficacy of manual stimulation of acupuncture points (manual acupuncture), electroacupuncture, bee venom acupuncture or any combination of the above. This scarcity of literature is similarly reflected in human studies; a recent systematic review on lumbar disc herniation (Tang et al., 2018) described only one study that compared manual acupuncture and a combination of middle frequency electrotherapy plus traction and exercises. That study showed an unfavourable effect of acupuncture when it was compared to the combination of treatments, but the language of the text was not written in English (conclusion was drawn from the systematic review (Tang et al., 2018) rather than the study it references (Shi, 2013)). Overall, it is unclear if there are any specific advantages of the different variations of acupuncture treatments.

Both Han et al. (2010) and Hayashi et al. (2007) used a combination of traditional Chinese acupuncture and electroacupuncture. It is unclear why this combination was chosen as opposed to solely using electroacupuncture. Lindley & Cummings (2006) state that with regards to electrotherapy, low-frequency stimulation, ranging from 2–15 Hz, mirrors the frequency of stimulation achieved through manual acupuncture and was used in this manner in the past to reduce the number of acupuncturists required per procedure.

Tsai et al. (2015) used bee venom injections in acupuncture points, this treatment has been described across a range of veterinary species and conditions (Chen et al., 2014; Jun et al., 2008; Kang et al., 2011; and Kang et al., 2012). As such, the author of this Knowledge Summary felt this acupuncture variant necessitated inclusion in this Knowledge Summary due to its documented frequent use in veterinary acupuncture.

Two clinical decisions the author of this Knowledge Summary felt should be highlighted was firstly, the concurrent use of carprofen and prednisone by Tsai et al. (2015); the combination of a corticosteroid and a nonsteroidal anti-inflammatory drug (NSAID) has been shown in a canine study (Narita et al., 2007) to significantly increase the risk of gastroduodenal ulceration and erosion, which in turn is potentially linked to death, as shown in another canine study (Pavlova et al., 2021). Secondly, the lack of pain management in these studies falls below the minimum standard of care expected in veterinary medicine; and the use of prednisolone is unjustified in the treatment of IVDE, according to the medical management guidelines written by Olby et al. (2022).

In conclusion, the lack of a unified validated scale, uncertainty regarding the timings of outcome assessment, increased risk of type 1 error, not having sufficiently large sample sizes, utilisation of prednisone instead of NSAIDs for medical management of IVDE without appropriate analgesia and basing eligibility criteria on tests with low to moderate sensitivities thus having an absence of a clear IVDE diagnosis, all raise concerns about the papers and consequently minimise the validity of the studies’ outcomes. However, as there are two prospective papers and one retrospective paper that all support the same conclusion, the strength of evidence is still moderate.

As mentioned above, the process of selecting papers for this Knowledge Summary was a challenge due to the scarcity of adequately published studies in this specific field. Furthermore, the selected studies exhibited substantial limitations, emphasising the need for further comprehensive research to provide more robust evidence in this area.

In a clinical scenario, when presented with an nonambulatory dog with suspected IVDE but referral is not an option, the clinician could consider offering a combination of electroacupuncture and acupuncture as an adjunct treatment, especially since it is unlikely to cause any harm and may be associated with a better outcome in these patients. However, the prioritisation in terms of treatment should still be for established medical management protocols.

## Methodology

**Search Strategy:** 

Databases searched and dates covered:	CAB Abstracts on CAB Direct from 1973 to 2 February 2024 PubMed on NCBI interface from 1975 to 2 February 2024
Search strategy:	CAB Abstracts: (Dog OR dogs OR “Canis familiaris” OR “Canis lupus familiaris” OR cani*) (Disc* OR disk* OR vertebra* OR spinal OR spine OR myelopath*) (Displacement* OR Protrusion* OR protruded OR Hernia* OR Slipped OR Prolapse* OR Degenerati* OR Degradation* OR Disease* OR Extrusion* OR Lesion* OR Disorder* OR pathology OR compression* OR injury OR injuries OR stenos*) (Acupuncture OR Pharmacoacupuncture OR Pharmacopuncture OR Acupotom* OR Electroacupuncture* OR electro-acupuncture OR Auriculotherapy OR "auricular therapy" OR "auriculoacupuncture") 1 and 2 and 3 and 4 Pubmed: (((Dog OR dogs OR "Canis familiaris" OR "Canis lupus familiaris" OR cani*) AND (Disc* OR disk* OR vertebra* OR spinal OR spine OR myelopath*)) AND (Displacement* OR Protrusion* OR protruded OR Hernia* OR Slipped OR Prolapse* OR Degenerati* OR Degradation* OR Disease* OR Extrusion* OR Lesion* OR Disorder* OR pathology OR compression* OR injury OR injuries OR stenos*)) AND (Acupuncture OR Pharmacoacupuncture OR Pharmacopuncture OR Acupotom* OR Electroacupuncture* OR electro-acupuncture OR Auriculotherapy OR "auricular therapy" OR "auriculoacupuncture")
Dates searches performed:	02 Feb 2024

**Exclusion / inclusion criteria:** 

Exclusion:	Not relevant to the PICO. Review article. Single case report / case series. Paper about cervical disc disease. In a language other than English.
Inclusion:	Randomised controlled trial. Cohort study.

**Search outcome:** 

Database	Number of results	Excluded – Not relevant to the PICO	Excluded – Review article	Excluded – Single case report / case series	Excluded – Paper about cervical disc disease	Excluded – In a language other than English	Total relevant papers
CAB Abstracts	103	78	16	7	1	1	0
PubMed	59	25	22	5	1	3	3
Total relevant papers when duplicates removed							3

## Acknowledgements

The author would like to thank Dr. Jo Ireland from the University of Liverpool for answering all the questions the author had when creating the Knowledge Summary.

## ORCiD

Justin Ng: https://orcid.org/0009-0001-3552-5232

## Conflict of interest

The author declares no conflicts of interest.

## Disclaimer

Knowledge Summaries are a peer-reviewed article type which aims to answer a clinical question based on the best available current evidence. It does not override the responsibility of the practitioner. Informed decisions should be made by considering such factors as individual clinical expertise and judgement along with patient’s circumstances and owners’ values. Knowledge Summaries are a resource to help inform and any opinions expressed within the Knowledge Summaries are the author's own and do not necessarily reflect the view of the RCVS Knowledge. Authors are responsible for the accuracy of the content. While the Editor and Publisher believe that all content herein are in accord with current recommendations and practice at the time of publication, they accept no legal responsibility for any errors or omissions, and make no warranty, express or implied, with respect to material contained within. For further information please refer to our Terms of Use.
